# Simultaneous Compression of the Neurovascular Bundle of Both Arms by a Bilateral Supracondylar Humeral Process: A Rare Case of Bilateral Nerve Entrapment of the Elbow in a Child

**DOI:** 10.7759/cureus.22694

**Published:** 2022-02-28

**Authors:** Byron Chalidis, Eleni Karagergou, Panagiotis Givissis

**Affiliations:** 1 First Department of Orthopedics, School of Medicine, Aristotle University of Thessaloniki, Thessaloniki, GRC; 2 Department of Burns, Plastic Surgery and Hand Surgery, George Papanikolaou General Hospital, Thessaloniki, GRC

**Keywords:** bone spur, struthers ligament, supracondylar process, brachial artery, median nerve

## Abstract

The supracondylar process is a beak-shaped bone spur arising from the anteromedial area of the distal humerus and in the majority of cases, it is connected to the medial epicondyle with a band of connective tissue which is known as ligament of Struthers. The complex of bone spur and ligament creates a ring that may compress the median nerve causing soreness and paresthesia of the hand and fingers. We present a rare case of bilateral supracondylar process compressing the neurovascular bundles in both arms and causing simultaneous bilateral upper limb pain, numbness, and weakness in an otherwise healthy young child. Surgical excision of the bone spurs and release of Struthers' ligaments provided immediate pain relief and complete resolution of symptoms. Three years after the index surgery, no limitations of activities or signs of recurrence were reported. Median nerve compression neuropathy in a child or a young adult should always raise the suspicion of the presence of supracondylar process particularly when bilateral symptoms exist. Although there are limited data about the incidence of recurrence and the extent of bone resection, the supracondylar process should be excised together with the overlying periosteum to minimize the theoretical risk of local regrowth.

## Introduction

The supracondylar process is a well-documented anatomical variant with an incidence between 0.4% and 2.7% [1-3]. It is the embryologic vestigial remnant of the supracondylar foramen, and it is more frequently encountered by Caucasians than in other ethnic groups [3,4]. The supracondylar process is a beak-shaped bone spur emerging from the distal anteromedial humerus and approximately 4-8 cm proximal to the medial epicondyle [5,6]. In almost all cases, it is connected to the medial epicondyle with a band of connective tissue which is known as the ligament of Struthers. The latter may be ossified and abnormally connected to pronator teres and coracobrachialis muscles [7]. The complex of spur and ligament creates a ring that compresses the median nerve, particularly during elbow extension, forearm pronation, and similar activities [6,7]. Therefore, clinical signs of pain, numbness, tingling, paresthesia, and weakness in the distribution of median nerve may be encountered. Here, we present a rare case of simultaneous bilateral compression of the arm neurovascular bundle in a healthy young child due to bilateral supracondylar process that resulted in significant and progressive symptoms in both upper limbs.

## Case presentation

**Figure 1:**
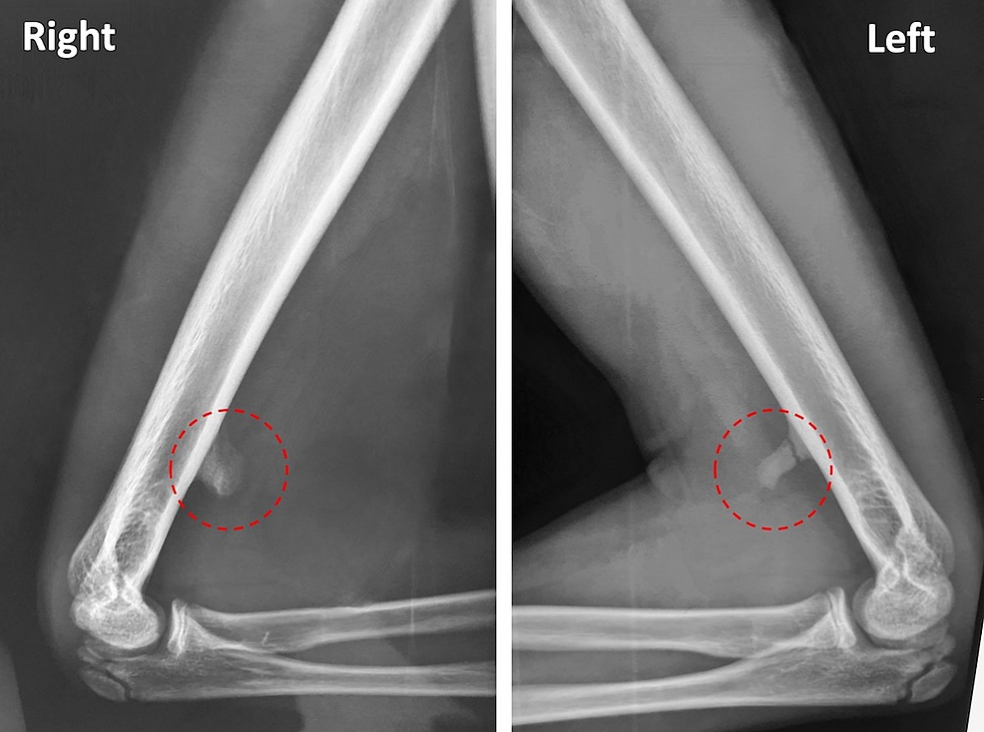
Bilateral elbow radiographs demonstrated the supracondylar process at the anterior side of the distal humerus. A radiolucent line was evident in the base of the left bone spur that indicated a stress fracture or incomplete fusion of the supracondylar process.

A 12-year-old Caucasian male patient without previous trauma and negative medical history presented with a six-month history of progressive, subacute, burning pain in the volar aspect of his both distal arms and along the distribution of median nerve. Symptoms were initially apparent during sports and manual exertive activities but gradually were present at rest and even during the night. The patient also reported paresthesia and numbness of the hands and involved fingers during the night hours. On physical examination, there was marked tenderness at palpation on both anteromedial supracondylar arm regions and a Tinel sign was elicited on gentle percussion of the area bilaterally. No motor or sensory deficit was observed, and electromyography did not reveal any specific abnormality. Radiographs showed an anteromedial supracondylar bone spur in both humeri approximately 6 cm from the medial epicondyle. A radiolucent line was evident in the base of the left bone spur that indicated a stress fracture or incomplete fusion of the supracondylar process (Figure 1).

**Figure 2:**
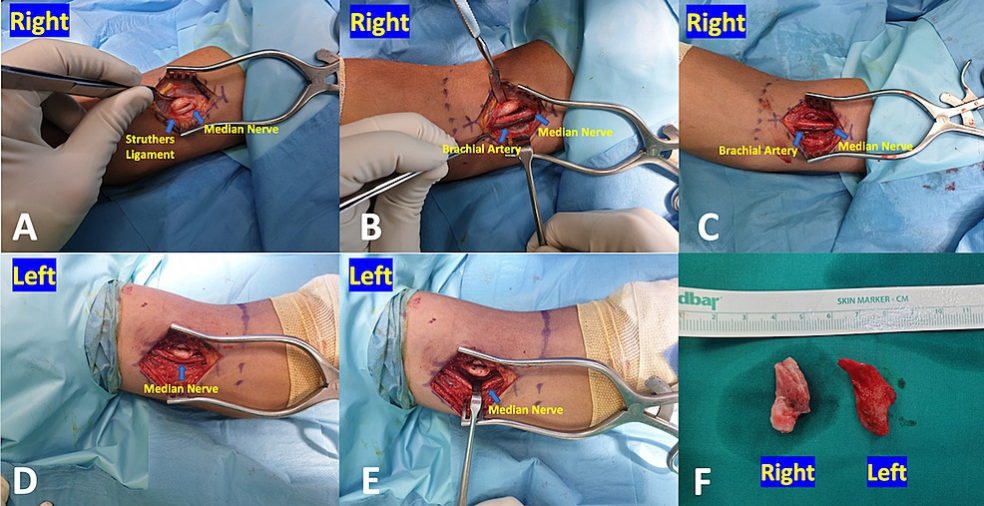
Intraoperative view of (A, B) right distal humerus shows the bone spur and Struthers ligament compressing the median nerve and brachial artery; (C) released neurovascular bundle after removal of bone exostoses; (D, E) the left elbow shows the supracondylar process to cover and compress the median nerve; (F) the shape and size of both excised bone spurs.

Under general anesthesia, a bilateral anteromedial approach of the distal humerus was applied. On both sides, the median nerve along with the brachial artery and vein were recognized at the bicipital medial sulcus and in close proximity with the bone spur. The dense fibrous band (ligament of Struthers) derived from the exostoses and running distally was also recognized superficial to the neurovascular bundle which was found compressed and slightly deformed. Both osteocartilaginous exostoses were excised from their base with an osteotome and the attached Struthers' ligaments were also resected to release the median nerves and the adjacent brachial arteries (Figure 2). 

Bone wax was not utilized to avoid the possibility of infection and foreign body reaction. Histopathological examination confirmed the benign character of the bone spurs, excluding malignancy. The postoperative course was uneventful, and the patient experienced immediate pain relief and complete resolution of symptoms. Three years after the index surgery, no limitations in his activities or signs of recurrence were reported. The disabilities of the arm, shoulder, and hand (DASH) score were zero in both upper limbs.

## Discussion

Bilateral median nerve neuropathy due to compression by the supracondylar process and ligament of Struthers is rare and only few cases have been described so far [2,5,8,9]. Subasi et al. reported a young female patient with bilateral median nerve compression and pain in arms, forearms, and hands due to a supracondylar process [2]. However, the patient refused surgery and further follow-up was not arranged. Aydinoglou et al. presented a case of a 21-year-old woman suffering from median nerve neuropathy in both upper limbs [5]. The nerve was found to be compressed by the Struthers' ligament which was extended from a supracondylar bone eminence to the apex of the medial humeral epicondyle. The spur was excised together with the periosteum, and the ligament was resected to facilitate normal nerve glide and mobility. The patient felt instant pain relief after the operation and the symptoms were almost disappeared two weeks later. Al-Qattan and Husband reported bilateral compression of the median nerve from a supracondylar process in a 70-year-old woman [8]. However, the symptoms did not occur simultaneously but several years apart. In our case, pain and paresthesia in the distribution of median nerve appeared concomitantly in both upper limbs in a young child of 12-years-old. Similar to the previous cases, electromyography did not recognize any pathology and failed to reveal any neurological deficit. According to our knowledge, this is the first bilateral symptomatic case in a child in the literature.

Apart from compression of the median nerve, the pain may also have a vascular origin. Meda et al. presented a 35-year-old man with compression of the brachial artery and acute ischemia as well as distal embolization caused by a supracondylar process and abnormal high insertion of the pronator teres to the bone spur [7]. The authors performed a computed tomography angiogram which revealed compression of the brachial artery from the supracondylar spur and distal eccentric thrombus extending into radial and ulnar arteries. Thromboembolic occlusion of two digital vessels was also apparent. Likewise, we found deformation and compression of the brachial artery in both arms due to pressure from the supracondylar process and the Struthers' ligament, but no angiography was performed to confirm limb ischemia.

Fracture of the bone spur or concomitant compression of the ulnar nerve has been also described as a rare cause of pain in the presence of supracondylar process [3]. Pedret et al. reported a case of a professional tennis player suffering from non-traumatic anteromedial arm pain and elbow stiffness [10]. The symptoms were attributed to stress fracture of the supracondylar humeral process after excessive traction of the pronator teres and the diagnosis was confirmed with x-rays and MRI. In this scenario, a previous asymptomatic bone spur may cause acute and permanent symptoms from the neurovascular bundle and therefore surgical intervention is deemed necessary [3,11]. In our case, a radiolucent line was apparent in one of the two supracondylar processes that indicated a stress fracture or incomplete fusion as no injury was reported. However, we did not identify intraoperatively any excessive scar formation or fibrosis around the bone spur compared to the other side. Besides, May-Miller et al. presented a 33-year-old male suffering from locking of his right elbow and ulnar nerve compression neuropathy due to a supracondylar process [3]. Specifically, there was pain and paresthesia at the medial side of the forearm and along the ring and little fingers. During surgery, the ulnar nerve was found to be kinked and compressed by a fascial/muscular band extended from the apex of the bone spur to the olecranon. 

Surgical excision of the bone spur and release of Struthers' ligament are usually adequate to provide complete and permanent pain relief when conservative treatment fails to alleviate the symptoms [3,6]. Surgical exploration should be undertaken along the course of the neurovascular bundle to identify the structure responsible for nerve and/or brachial artery compression [11]. Although there are limited data about the incidence of recurrence and no consensus exists regarding the extent of bone resection, the supracondylar process should be excised together with the overlying periosteum to minimize the theoretical risk of local regrowth [3,7]. Opanova and Atkinson summarized 38 previously published supracondylar process syndrome cases that were treated operatively [12]. Pain relief was immediately achieved in days or at least within a couple of weeks and recurrence occurred only in two cases after surgery.

## Conclusions

In conclusion, a physician should be aware of the presence of supracondylar process in a healthy young child with upper limb pain, numbness, and weakness due to compression of median nerve and/or brachial artery. The symptoms may be bilateral and present simultaneously without a history of trauma or injury. Routine anteroposterior and lateral radiographs are adequate to visualize the pathology and define the diagnosis. Electromyography usually is normal and its value in recognizing the pathology is limited. Surgical excision of the bone spur and its associated Struthers' ligament with subsequent release of the median nerve and/or brachial artery is recommended when conservative treatment could not alleviate the pain and resolve the symptoms. Operative management is associated with prompt pain relief, full functional recovery, and low possibility of recurrence.
